# Solution and Crystallographic Structures of the Central Region of the Phosphoprotein from Human Metapneumovirus

**DOI:** 10.1371/journal.pone.0080371

**Published:** 2013-11-04

**Authors:** Cedric Leyrat, Max Renner, Karl Harlos, Jonathan M. Grimes

**Affiliations:** 1 Division of Structural Biology, University of Oxford, Oxford, United Kingdom; 2 Science Division, Diamond Light Source Ltd., Didcot, United Kingdom; University of Alabama at Birmingham, United States of America

## Abstract

Human metapneumovirus (HMPV) of the family *Paramyxoviridae* is a major cause of respiratory illness worldwide. Phosphoproteins (P) from *Paramyxoviridae* are essential co-factors of the viral RNA polymerase that form tetramers and possess long intrinsically disordered regions (IDRs). We located the central region of HMPV P (P_ced_) which is involved in tetramerization using disorder analysis and modeled its 3D structure *ab initio* using Rosetta fold-and-dock. We characterized the solution-structure of P_ced_ using small angle X-ray scattering (SAXS) and carried out direct fitting to the scattering data to filter out incorrect models. Molecular dynamics simulations (MDS) and ensemble optimization were employed to select correct models and capture the dynamic character of P_ced_. Our analysis revealed that oligomerization involves a compact central core located between residues 169-194 (P_core_), that is surrounded by flexible regions with α-helical propensity. We crystallized this fragment and solved its structure at 3.1 Å resolution by molecular replacement, using the folded core from our SAXS-validated *ab initio* model. The RMSD between modeled and experimental tetramers is as low as 0.9 Å, demonstrating the accuracy of the approach. A comparison of the structure of HMPV P to existing *mononegavirales* P_ced_ structures suggests that P_ced_ evolved under weak selective pressure. Finally, we discuss the advantages of using SAXS in combination with *ab initio* modeling and MDS to solve the structure of small, homo-oligomeric protein complexes.

## Introduction

Human metapneumovirus (HMPV) is a major cause of acute respiratory diseases in children, the elderly and immunocompromised patients worldwide [1–5]. HMPV belongs to the *Pneumovirinae* subfamily of the *Paramyxoviridae* and is further classified into the genus Metapneumovirus[6]. HMPV is an enveloped virus that forms pleomorphic or filamentous virions. Its genome consists of a ~13-kb single stranded RNA molecule of negative polarity that encodes 9 proteins in the order ^3^’-N-P-M-F-M2(-1)/(-2)-SH-G-L-^5^’. HMPV proteins show detectable levels of sequence identity to the respiratory syncytial virus (RSV) (genus *Pneumovirus*); however, the order of the genes is different and HMPV lacks the NS1 and NS2 genes present in RSV. For all paramyxoviruses, the nucleoprotein (N) encapsidates viral RNA, leading to a N-RNA complex which, together with the RNA-dependent RNA polymerase (L) and the phosphoprotein (P), forms the viral replication complex. The P protein is thought to be responsible for the recruitment of the large polymerase L onto the viral N-RNA template through direct interactions with N and L [7–15]. In addition, P chaperones the nascent N, which is sequestered in the form of an RNA-free NP complex [16,17]. The M2 gene is specific to the *Pneumovirinae* subfamily, and possesses two overlapping open reading frames encoding two proteins, the antitermination/transcription-elongation factor M2-1, which is required for viral transcription [18], and the RNA synthesis regulatory factor M2-2 [19].

For all members of the *Paramyxoviridae* family, the P protein is an intrinsically disordered polypeptide which forms tetramers through a central α-helical coiled-coil region. Available structures of the tetrameric coiled-coil from Sendai virus (SeV) [20] and Measles virus (MeV)[21] show long parallel arrangements of twisted α-helices. However, the structure of the Mumps virus phosphoprotein strikingly reveals the formation of parallel dimers that further assemble into tetramers by associating in an antiparallel fashion [22]. In contrast, the tetramerization domain of the RSV P protein, which is the closest homologue of HMPV to have been structurally characterized, displays a much shorter coiled-coil region, termed fragment Y*. This fragment has been previously identified via proteolytic digestion and consecutively mapped to residues 119 to 160 by mass-spectrometry and N-terminal sequencing [23,24]. Interestingly, although the length of the HMPV P sequence is greater than that of RSV P by 53 residues and the overall sequence identity is only 28%, conservation is considerably higher in the central structured region of the protein, suggesting similar tetramerization domains. 

In this study, we applied bioinformatics approaches to locate the central folded region of HMPV P, and used symmetric homo-oligomeric *ab initio* modeling in combination with small angle X-ray scattering (SAXS) and molecular dynamics simulations (MDS) to determine the structure of the central region of HMPV P (P_ced_) and capture its flexibility in solution. We used the obtained model to solve the crystal structure of the core region of P_ced_ (residues 168-194) by molecular replacement. We analyze the implications of the structure of P_ced_ for virus function and evolution, and discuss the usefulness of integrative approaches to protein structure determination. 

## Results

### Disorder analysis locates the central structured region of P_ced_

**Figure 1:**
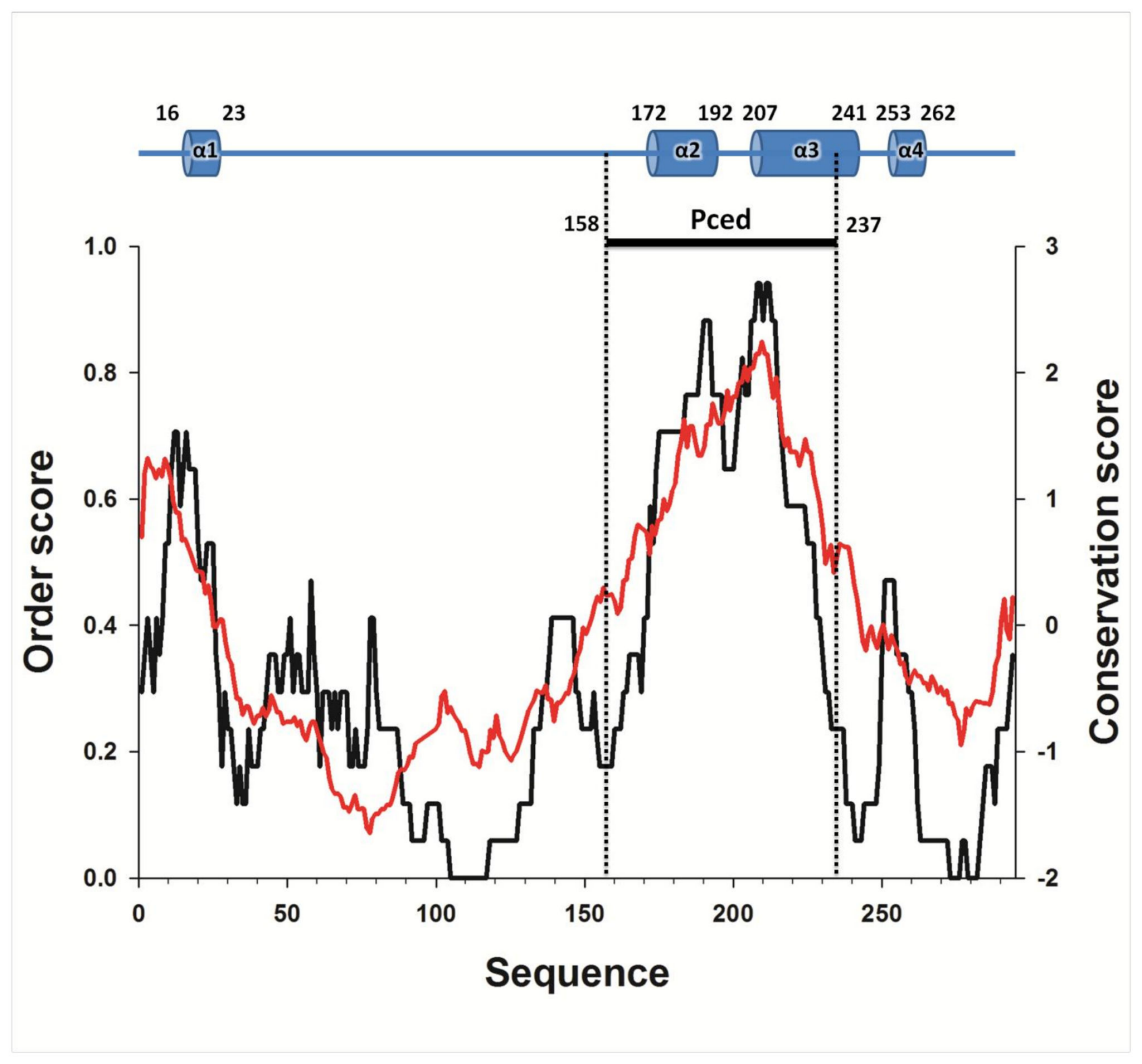
Sequence-based analyses of human metapneumovirus phosphoprotein. The predicted propensity to adopt ordered structures is represented along the amino-acid sequence (black line), together with the conservation score (red line), calculated using AL2CO [76]. The location of the predicted secondary structure elements and the identity of the cloned construct are shown above the graphs.

We used meta-disorder predictions in combination with sequence conservation and secondary structure propensity to locate IDRs and folded regions of HMPV P (Figure 1). The analysis predicts the presence of a central, highly conserved region with α-helical propensity located between residues 158 to 237, which we refer to as P_ced_. The N-terminal and C-terminal regions flanking P_ced_ are mostly disordered and weakly conserved, with the notable exception of the first 30 residues, which show a narrow peak of conservation and predicted order, suggesting the presence of an α-helical molecular recognition element (MoRE), as has been described for P proteins from other members of the *Paramyxoviridae* [16] and *Rhabdoviridae* families [25].

### Structural characterization of P_ced_ by SAXS

**Figure 2:**
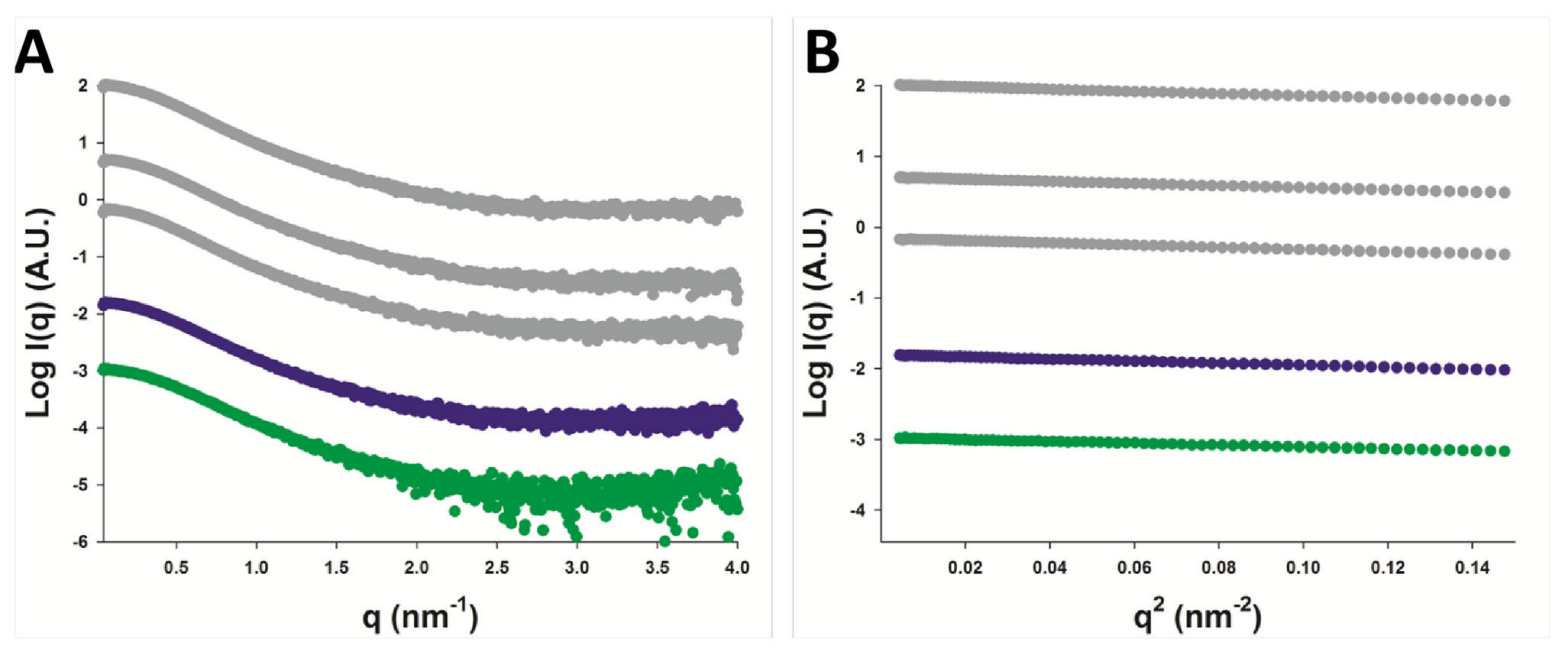
Small Angle X-ray Scattering experiments (SAXS). A. SAXS profiles of P_ced_ measured in the presence of 20 mM Tris pH 7.5, 150 mM NaCl at three different protein concentrations (4, 5 or 6 mg/ml) (grey spheres), in 20 mM Tris pH 7.5, 800 mM NaCl (purple spheres), or 20 mM Tris pH 7.5, 150 mM NaCl and 1M NDSB-201 (green spheres). B. Corresponding Guinier plots, showing linear behavior in the low q range.

P_ced_ was expressed and purified in *E.coli* and its structure was characterized using SAXS (Figure 2 A). The samples were free from aggregates, as evidenced by the linearity of the Guinier region (Figure 2 B). The parameters derived from SAXS data are summarized in Table 1. Radii of gyration (R_g_) were independent of protein concentration, and only moderately affected by salt concentration (R_g_ =3.26 ± 0.02 nm in 150 mM NaCl vs 3.17 ± 0.04 nm in 800 mM NaCl). However, a significant drop in R_g_ to a value of 2.98 ± 0.03nm was observed in the presence of 1M of non-detergent sulfobetaine 201 (NDSB-201), suggesting an induced stabilization of P_ced_ structure. Molecular weights (MW) were estimated based on calculation of the concentration-independent volume of correlation V_c_, as defined in[26], yielding values between 28 and 34 kDa, in agreement with the MW calculated from the amino-acid sequence, assuming a tetramer (8.8 x 4 = 35 kDa).

**Table 1:** SAXS-derived parameters.

**Buffer conditions**	**Concentration (mg/ml)**	**Molecular weight (kDa)**	**Radius of gyration (nm)**
20 mM Tris pH 7.5 150 mM NaCl	4	33	3.23 ± 0.05
20 mM Tris pH 7.5 150 mM NaCl	5	32	3.25 ± 0.04
20 mM Tris pH 7.5 150 mM NaCl	6	34	3.26 ± 0.02
20 mM Tris pH 7.5 300 mM NaCl 1M NDSB-201	3	30	2.98 ± 0.03
20 mM Tris pH 7.5 800 mM NaCl	3	28	3.17 ± 0.04

### Modelling of P_ced_ using Rosetta Fold-and-dock and SAXS-based model selection

**Figure 3:**
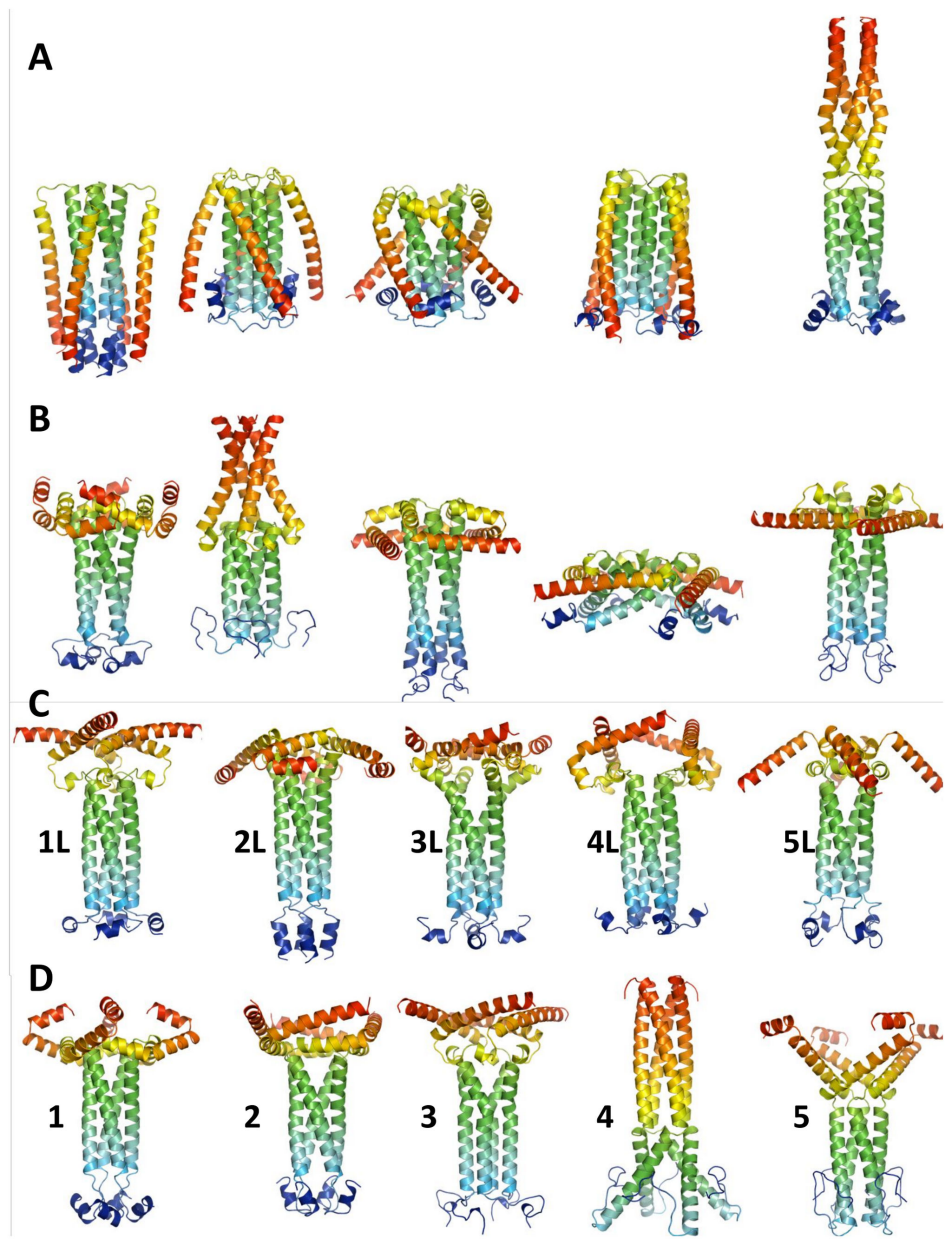
*Ab* *initio* models of P_ced_ before (A&B) and after (C&D) applying the SAXS filter. A. From left to right, the 5 best-scoring Rosetta fold-and-dock models generated from the sequence of HMPV P residues 155 to 241. Models are shown in cartoon and arecoloured from blue (N-terminus) to red (C-terminus). B. 5 best-scoring Rosetta fold-and-dock models from HMPV P residues 156 to 237. C and D. Same as in A and B after filtering out all models with χ_exp_> 1.3. All models were truncated to residues 156-237 to improve fitting accuracy (residues 156-157 were kept to account for the two extra N-terminal residues resulting from cleavage of the His6 tag by 3C protease).

We employed the Rosetta fold-and-dock application[27] to model the structure of P_ced_ tetramers. We generated 2 x 30,000 models using the sequence of residues 155-241 or 156-237. The use of two different sequence lengths leads to an increased structural diversity of sampled models, due to the fragment-based approach implemented in Rosetta. Moreover, the effects of possibly truncating the predicted α-helix (α3) located between residues 207-241 are taken into account (Figure 1). Both ensembles were ranked according to their Rosetta free energy score, and the five best models of each ensemble are shown in Figure 3 A and B. Interestingly, all models apart from a single oblate model, form a tetrameric coiled-coil through the arrangement of α-helices (α2) typically ranging from residues 168 to 198. We refer to this central region as P_core_. The regions comprising residues 158 to 168, and 200 to 237 display a tendency towards α-helical conformations, but adopt different orientations in each model, resulting in important changes to the overall shape of the predicted models. This lack of convergence suggests that these regions might not assume a single conformation in solution, but rather exist as disordered ensembles.

In a second step, we calculated theoretical SAXS profiles for all of the Rosetta ensembles and fitted them to the experimental SAXS profile, yielding an additional score for each model in the form of a χ_exp_ value, which measures the discrepancy between theoretical and experimental SAXS profiles. The best results were obtained using data measured in the presence of 1M NDSB-201, because of the increase in conformational stability it induces, as evidenced by the significant drop in R_g_ (Table 1). We filtered out all models displaying χ_exp_ values higher than 1.3, thus eliminating more than 90 % of the models and then ranked the remaining models according to their Rosetta score. The five best models from each ensemble are shown in Figure 3 C & D. Strikingly, we observe that the experimental SAXS profile imposes strong shape constraints on the models, leading to a more homogeneous ensemble (Figure 3C&D opposed to Figure 3A&B). With the exception of an oblate model which ranked third (not shown) and model 4,all models display a relatively similar coiled-coil arrangement of α-helices encompassing residues 168 to 198 (P_core_), while residues 158-167 and residues 199-237 adopt various, mostly α-helical structures.

### MDS confirm the stability of P_core_ and the flexibility of the flanking regions

**Figure 4:**
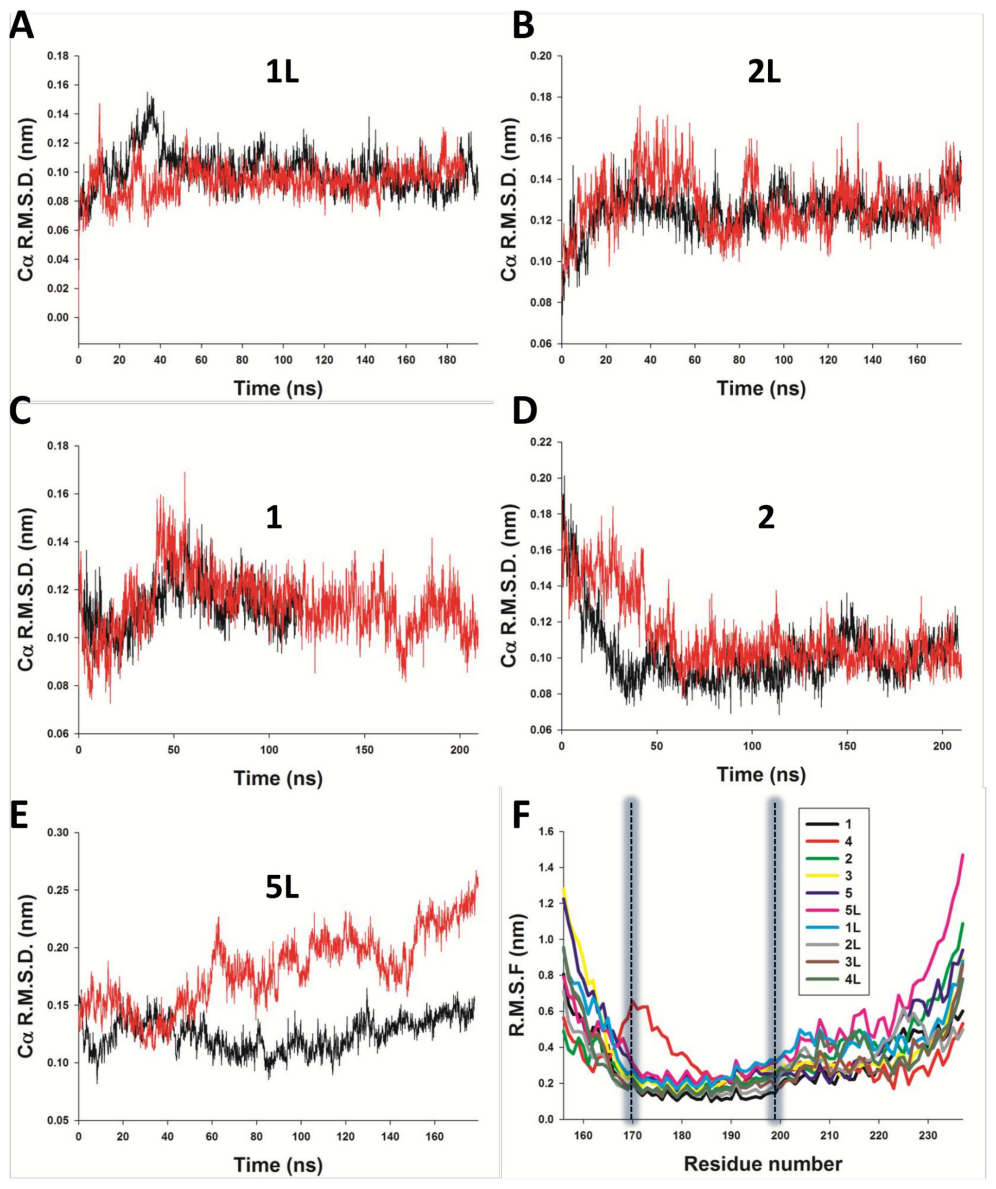
Molecular Dynamics simulations (MDS). A. Plot of root mean square deviation (RMSD) of model 1L P_core_ Cα atoms versus simulation time, with respect to the starting structure coordinates. B, C, D and E. RMSD of model 2L, 1, 2 and 5L P_core_ Cα atoms using model 1L P_core_ as reference structure. F. Time- and residue-averaged root mean square fluctuations (RMSF) of all simulated models (1L to 5L and 1 to 5). The displayed curves represent average RMSF values over the four chains of a tetramer and two independent simulations.

We tested the stability of the 10 best-fitting *ab initio* models via classical explicit-solvent molecular dynamics simulations (MDS), by performing duplicate runs of approximately 200 ns. The analysis of root mean square fluctuations (RMSF) along the sequence confirms the stability of the coiled coil region, with RMSF values centred around 2 Å for the P_core_ region. The flanking regions (residues 158-168 and 200-237) display higher RMSFs, indicating instability of the packing in most models (Figure 4 F), and further supporting the hypothesis that these residues are flexible in solution. Additionally, model 4, which displays a different P_core_ structure, is readily identified as an outlier due to its higher flexibility in the P_core_ region. The RMSD of P_core_ was calculated with respect to the starting structure, showing that model 1L displays the most stable P_core_ structure (Figure 4 A). P_core_ from model 1L was then used as a reference structure to study the conformational behavior of the other models during MDS (Figure 4 B, C, D and E). Models 1 and 2L fluctuated within about 1.0-1.4 Å RMSD of model 1L P_core_, showing that they adopt a similar structure (Figure 4 B&C). Interestingly, model 2 was also seen to converge within 1.0 Å RMSD of model 1L P_core_ after 50 ns of MDS, following an initial drop of 0.8 Å (Figure 4 D). Only model 5 P_core_ diverged significantly by more than 2.5 Å in one simulation (Figure 4 E). 

### SAXS-based ensemble analysis captures the dynamic character of P_ced_ in solution

**Figure 5:**
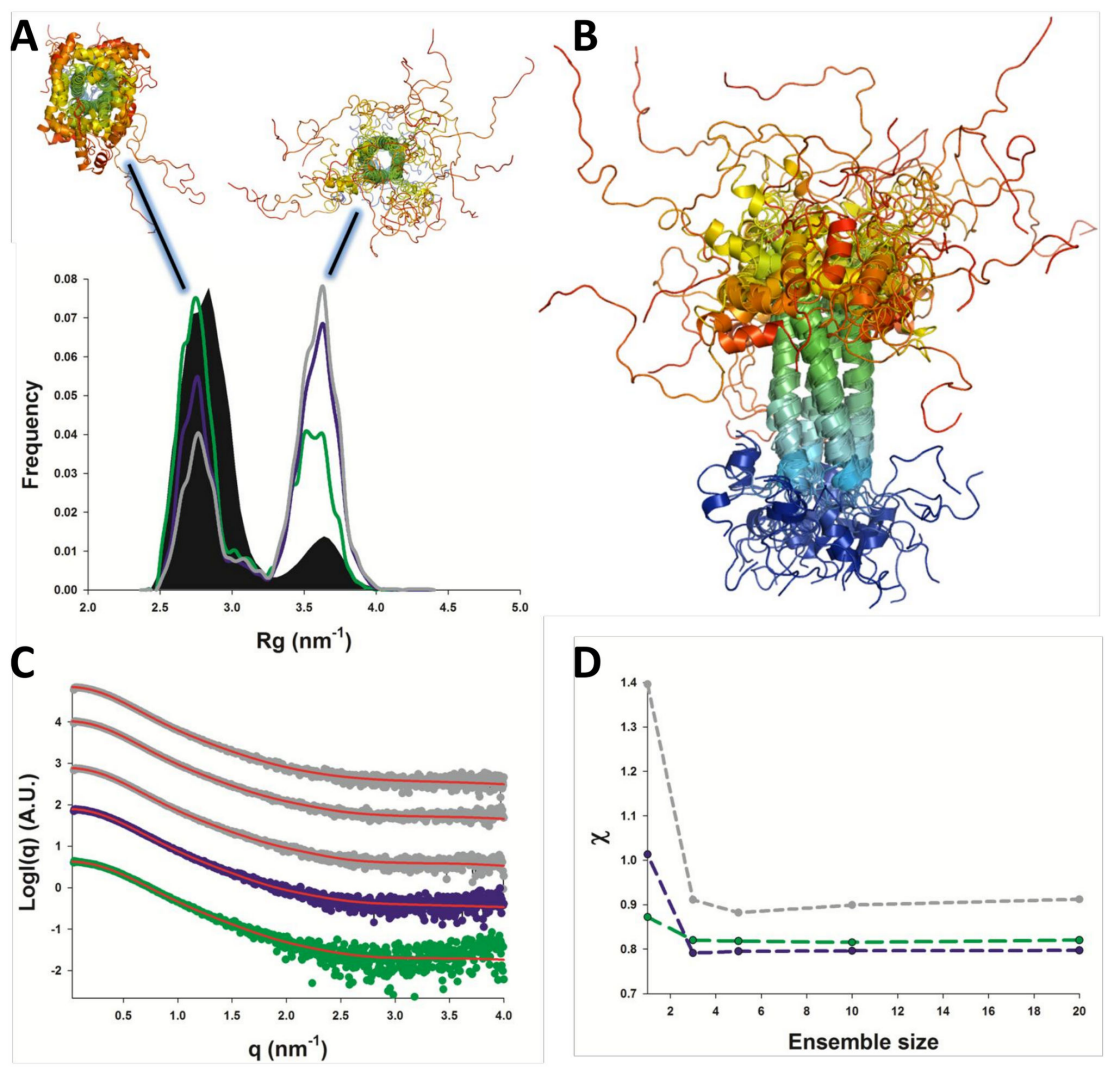
SAXS-based ensemble structure of P_ced_. A. Radius of gyration (R_g_) distributions of the pool (black area) and optimized ensembles selected in the presence of 150 mM NaCl (grey line), 800 mM NaCl (purple line) and 1 M NDSB-201 (green line). Representative conformers populating the two peaks of the R_g_ distribution are shown above the graph in cartoon representation and coloured from blue (N-terminus) to red (C-terminus). B. An ensemble of 10 conformers selected against the NDSB-containing sample, highlighting the dynamic equilibrium between random coil and α-helical conformations of the C-terminal region. C. EOM-fitted SAXS profiles of P_ced_ are represented by red lines. The colour-coding for the experimental curves is the same as in Figure 2. D. Variation of χ_exp_ with optimized ensemble size for SAXS profiles of P_ced_ measured in the presence of 20 mM Tris pH 7.5, 150 mM NaCl (grey line), in 20 mM Tris pH 7.5, 800 mM NaCl (purple line), or 20 mM Tris pH 7.5, 150 mM NaCl and 1M NDSB-201 (green line).

The time-averaged R_g_ of all simulated models ranges from 2.70 to 2.98 nm (Table 2),which is lower than the measured R_g_ for P_ced_ (3.26 nm) by at least 3 Å, clearly indicating that P_ced_ possesses IDRs that are not adequately modeled in our classical MDS. Interestingly, data measured in the presence of 1M NDSB-201 shows a R_g_ of 2.98 nm, suggesting a more stable fold of P_ced_ in these conditions, consistently with the lower χ_exp_ values observed for fitting of the Rosetta models (not shown). In order to explicitly model the flexibility of P_ced_ in solution, we employed the ensemble optimization method (EOM) [28]. To take into account the possibility of IDRs outside P_core_, we used atomistic structure-based models (SBM) [29] as a mean to rapidly sample the conformational space of residues 158-167 and 199-237 and impose extended or α-helical conformations. We then pooled all models from classical MDS and SBM MDS into an ensemble of ~ 12,300 models, which we fitted against SAXS data using EOM [28]to yield optimized ensembles (Figure 5). A representative ensemble of ten conformers is shown in Figure 5 B. SAXS profiles could be adequately fitted using models from the pool ensemble, with χ_exp_ values reaching a plateau at 0.8-0.9 for an ensemble size of 3 to 5 models (Figure 5 C and D), providing direct evidence for the presence of flexible regions in P_ced_ [30]. Interestingly, the drop in χ_exp_ value upon increasing ensemble size was less pronounced for the NDSB-containing sample, confirming the induced increase in protein fold stability, as has been observed previously [31]. The R_g_ distributions of the optimized ensembles measured in low or high salt conditions, or in the presence of 1M NDSB-201 are shown in Figure 5 A, revealing a dynamic equilibrium between two populations that correspond to the presence or absence of α-helices outside the core region. Interestingly, the addition of NDSB-201 correlated with an increase in the percentage of α-helices in the optimized ensemble (Figure 5 A). 

**Table 2:** summary of classical Molecular Dynamics simulations.

**Model**	**Time-averaged χ_exp_**	**Time-averaged Rg (nm)**	**System size (atoms)**	**Total simulation time (µs)**
**1**	**1.93**	**2.70**	**60814**	**0.32**
**2**	**1.37**	**2.87**	**58230**	**0.42**
**3**	**1.28**	**2.95**	**57043**	**0.51**
**4**	**1.16**	**2.98**	**64080**	**0.41**
**5**	**1.55**	**2.83**	**64641**	**0.40**
**1L**	**1.31**	**2.92**	**71871**	**0.38**
**2L**	**2.34**	**2.75**	**67503**	**0.36**
**3L**	**1.72**	**2.79**	**57138**	**0.41**
**4L**	**1.92**	**2.85**	**56027**	**0.42**
**5L**	**1.28**	**2.90**	**81087**	**0.36**

### Crystal structure of P_core_

**Figure 6:**
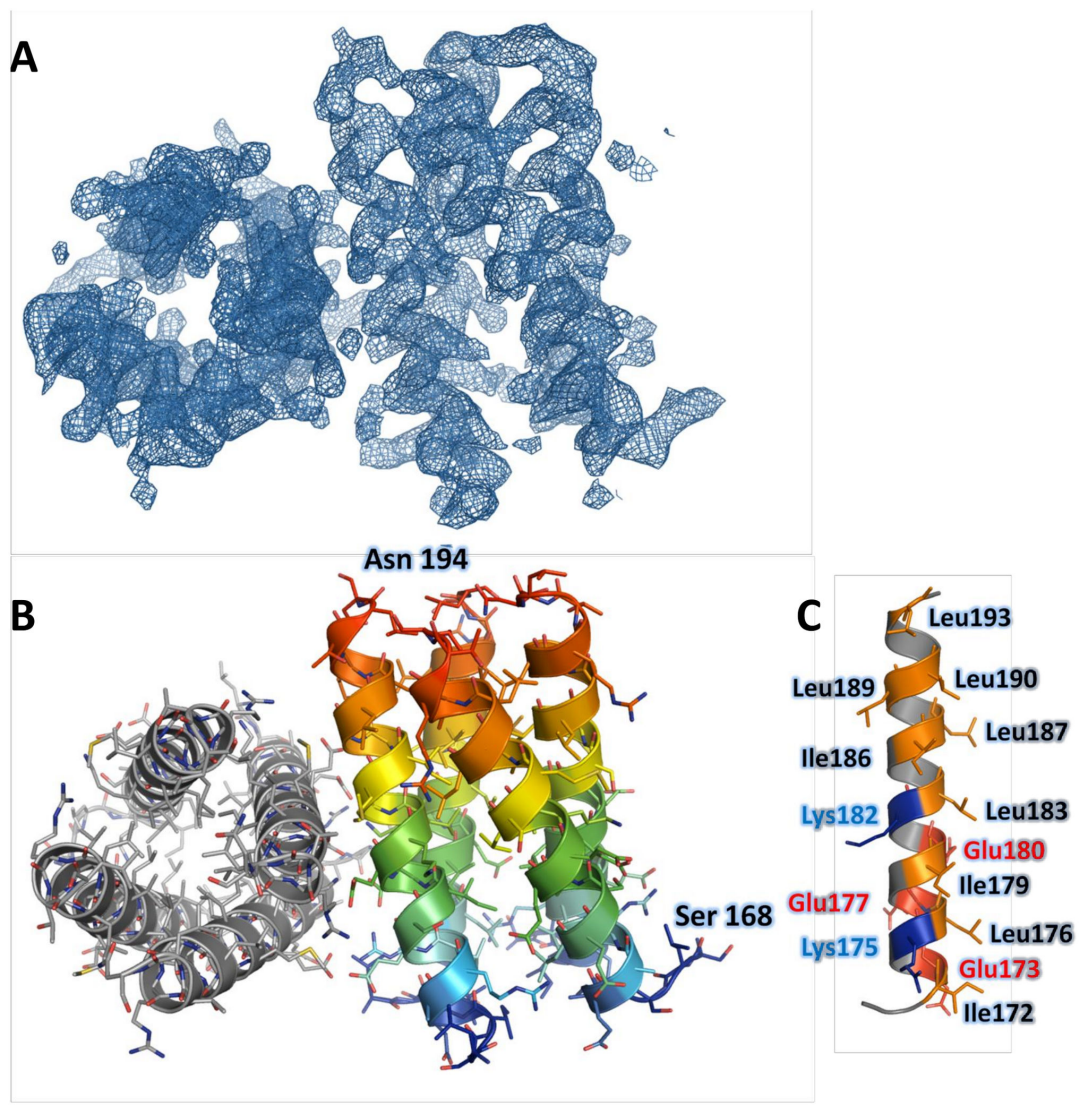
Crystal structure of P_core_. A. Isomesh surface of the experimental 2F_o_ - F_c_ electron density contoured at 1.4σ. B. Structure of the asymmetric unit, showing the close packing of two tetrameric molecules. The left tetramer is shown in grey, and the right one is coloured from blue (N-terminus) to red (C-terminus). C. Structure of a single subunit from the crystal, highlighting the residues involved in intermolecular contacts. Hydrophobic residues are coloured in orange, while positively and negatively charged residues are coloured in blue and in red, respectively.

**Figure 7:**
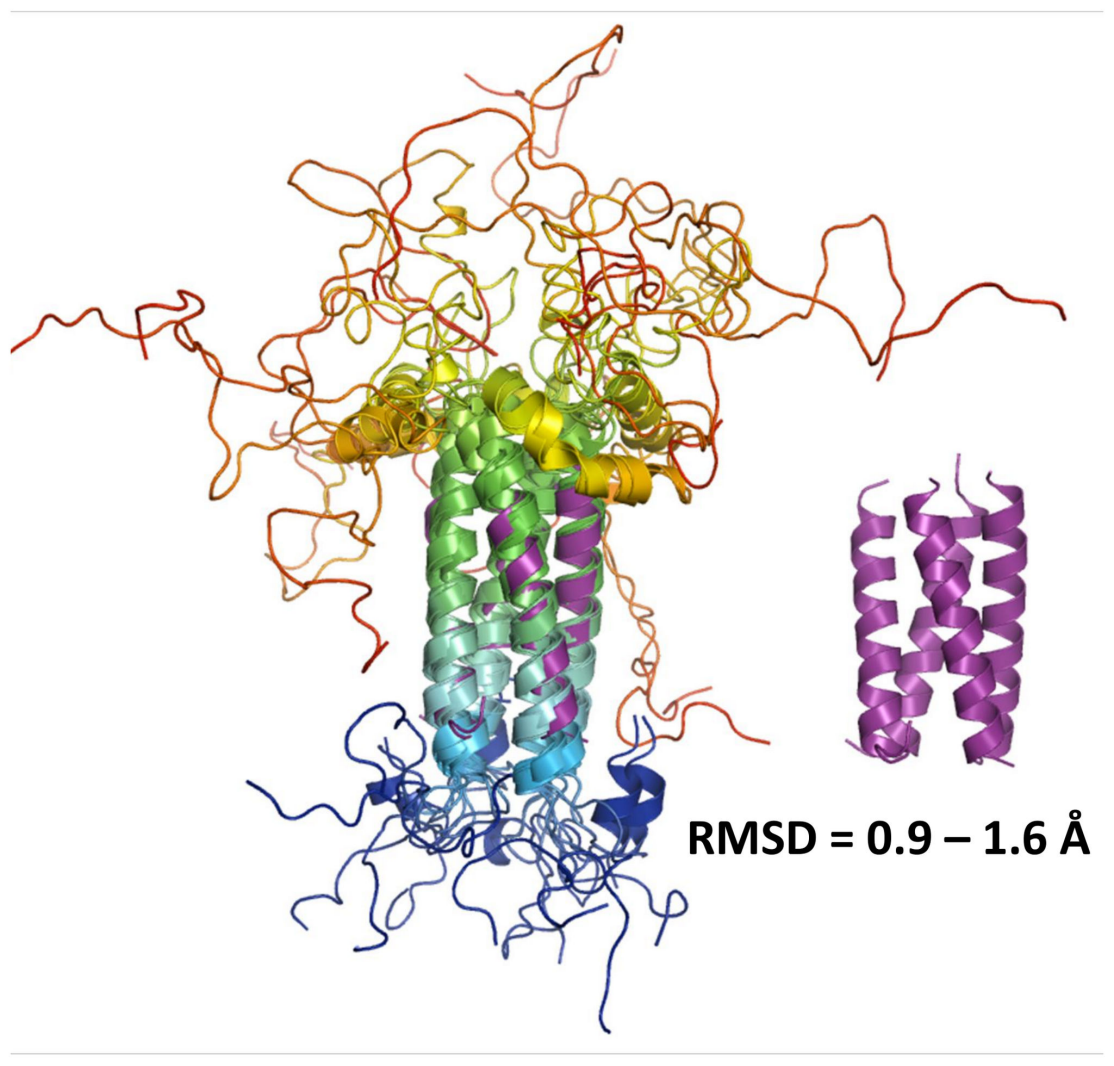
Comparison of the crystal structure of P_core_ with a minimal structural ensemble of P_ced_. Left panel, the crystal structure from P_core_ (purple cartoon) is overlayed with an optimized ensemble of 5 models of P_ced_ (coloured from blue (N-terminus) to red (C-terminus)) selected from SAXS data measured in 20 mMTris pH 7.5, 150 mMNaCl. Right panel, the crystal structure is additionally shown in purple cartoon. The observed range of Cα-RMSD values is indicated (calculated in Pymol).

A single diffracting crystal of HMPV P_ced_ grew after 140 days, suggesting degradation occurred in the drop prior to crystallization. The crystal belonged to space group P2_1_2_1_2_1_ (Table 3). Diffraction data were phased by molecular replacement using residues 169 to 194 from P_core_, revealing a tetrameric α-helical arrangement consistent with the modeled structure (Figure 6 A and B). The asymmetric unit contains 2 tetramers, with a solvent content of 40%. Since the asymmetric unit could not physically contain 2 tetramers with residues 158-237 (with 1 tetramer present the solvent content is 16%), packing considerations suggest that degradation necessarily occurred prior to crystallization. Model 1 was selected for molecular replacement because it displayed the lowest RMSF in the core α-helical region during MDS (Figure 4F). The stability of the P_core_ region in *ab initio* models, MDS and SAXS prompted us to use residues 168 to 198 as a search model, however this resulted in rejection of a potential solution with a high translation function Z-score (TFZ) due to steric clashes between tetramers, which were eliminated by using a shorter fragment encompassing residues 169 to 194. The structure was subsequently refined to R_work_ and R_free_ values of 23.5% and 25.2%, respectively, confirming the identity of the modeled residues. The residues 158-168 and 195-237 are absent from the structure, in line with the flexibility observed for these regions by MDS and SAXS-based ensemble optimization. The tetrameric structure is stabilized by a large network of hydrophobic interactions involving Leu176, 183, 187, 189, 190, 193, and Ile172, 179, 186 located on the inner surface of symmetry-related α-helices (Figure 6 C). Additional stability is provided by a solvent-exposed network of ionic interactions created by Glu173/Glu177 and Arg175, or Glu180 and Lys182 side chains from neighbouring protomers. Comparison with representative models from the SAXS optimized ensembles, as well as with the best scoring models from MDS (Figure 3 C & D) demonstrates Cα RMSDs ranging from 0.9 to 1.6 Å (Figure 7) over aligned residues, confirming the accuracy of the modeled P_core_. 

**Table 3:** Crystallographic statistics.

**Space group**	**P2_1_2_1_2_1_**
**Wavelength (Å)**	0.92
**Unit cell constants**	a=66.9 Å b=48.5 Å c=64.5 Å
**Resolution limits^a^**	66.9-3.1 Å (3.2-3.1 Å)
**Number of measured Reflections**	37981
**Number of unique Reflections**	3602
**Completeness of data^a^**	90.3% (57.1%)
**Rmerge^a^**	35.1% (134.7%)
**Rpim^a^**	15.7% (86.9%)
**Multiplicity^a^**	10.5 (6.1)
**I/σ^a^**	6.4(1.4)
**R_work_**	23.5%
**R_free_**	25.2%
**Number of residues in favoured region**	177 (96.7%)
**Number of residues in allowed region**	2 (1.1%)
**Number of residues in outlier region**	4 (2.2%)
**RmsBondLength**	0.010
**RmsBondAngle**	1.290

^a^ The values for the highest resolution shell are given in parentheses.

## Discussion

### The structure of P_ced_ reveals the shortest tetrameric coiled-coil among the Paramyxoviridae

**Figure 8:**
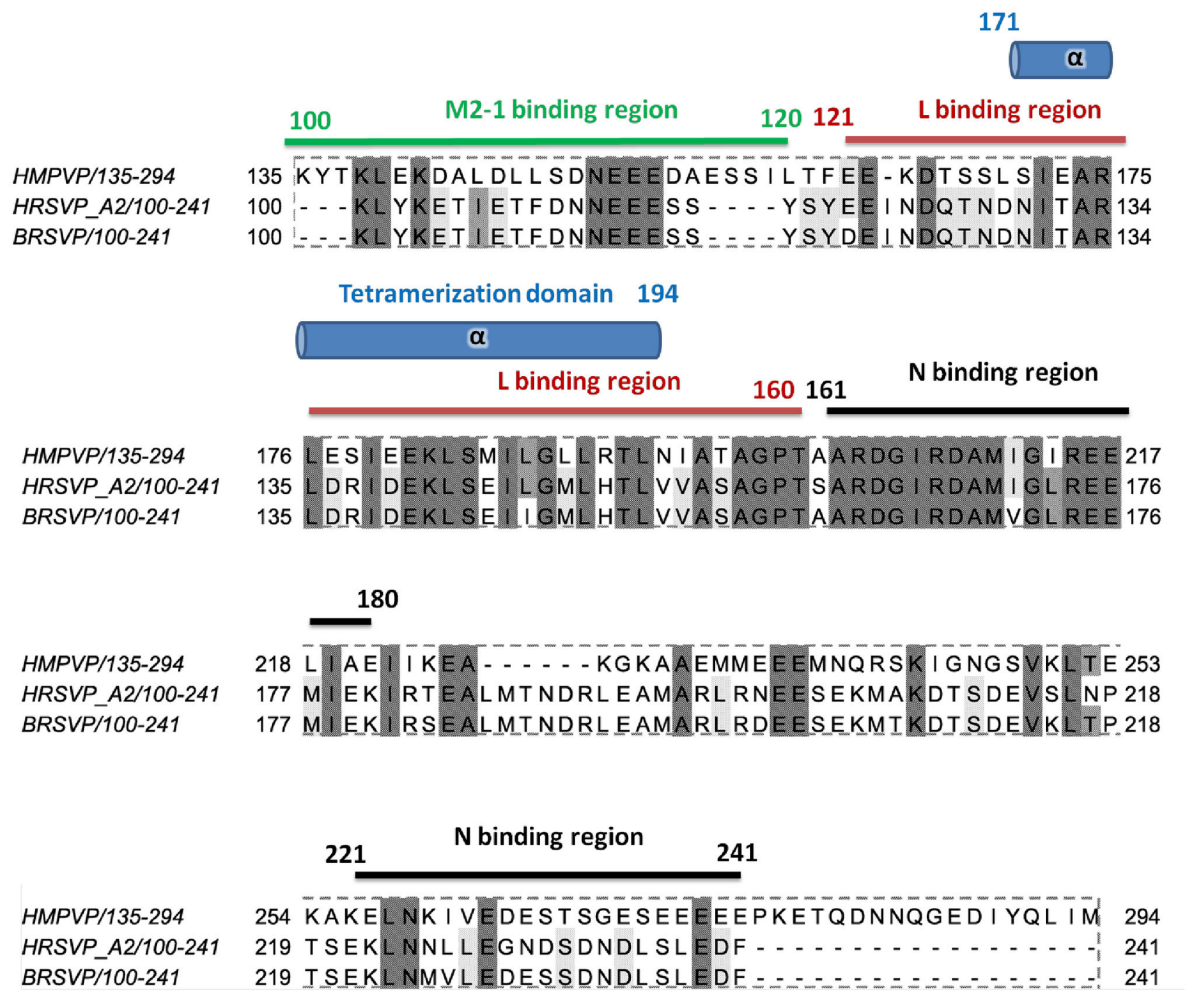
Annotated sequence alignment of the conserved C-terminal region of HMPV, HRSV and BRSV P. The location of the tetramerization domain (P_core_) and the position of conserved residues are highlighted. Regions that have been associated with N, M2-1 or L interaction in RSV are indicated by a bar based on [7,9,11,14,32].

The structural data presented here indicates that the α-helical tetramerization domain of HMPV P (residues 171-194) is considerably shorter than the highly conserved central region of the molecule (residues 158-237). Interestingly, the region 195-237 is shown by SAXS-based ensemble optimization to form an IDR with strong α-helical propensity. These transiently folded α-helices can be further stabilized by addition of NDSB-201. Taken together, these features suggest that residues 195 to 237 might constitute a molecular recognition element (MoRE) located directly downstream of the coiled coil region. A sequence alignment of HMPV P with HRSV and BRSV P is shown in Figure 8. The regions that have been mapped in RSV to be required for interaction with the N, L and M2-1 proteins are annotated based on published mutagenesis studies [7,9,11,14,32]. Interestingly, the sequence of P_core_ aligns with a region of RSV P that is necessary for coimmunoprecipitation of the L protein [11], suggesting either direct binding to L or the requirement of a tetrameric P protein for efficient P-L association. Additionally, the α-helical MoRE located between HMPV P residues 195 to 237 shows strong conservation and overlaps with a putative nucleoprotein (N) binding region identified in RSV (residues161-180). Residues 221 to 241 of RSV P are also part of a putative N binding site, which aligns with residues 257 to 277 of HMPV featuring the predicted α4 helix (Figure 1 and 8). The flexibility of α3 (residues 195 to 237) relative to α2 (P_core_) observed in the SAXS ensembles, together with the requirement of α3 and α4 for N binding, suggest that these regions may be part of a nucleoprotein-binding domain (NBD), similar to the C-terminal domain of phosphoproteins from other *Mononegavirales* [33–37]. Alternatively, the C-terminal region of HMPV P may act as a MoRE that folds upon binding to N, and assume an unstable tertiary structure in the absence of binding partner. This hypothesis is supported by the low propensity to form ordered structures observed for the C-terminal region of HMPV P (Figure 1).

**Figure 9:**
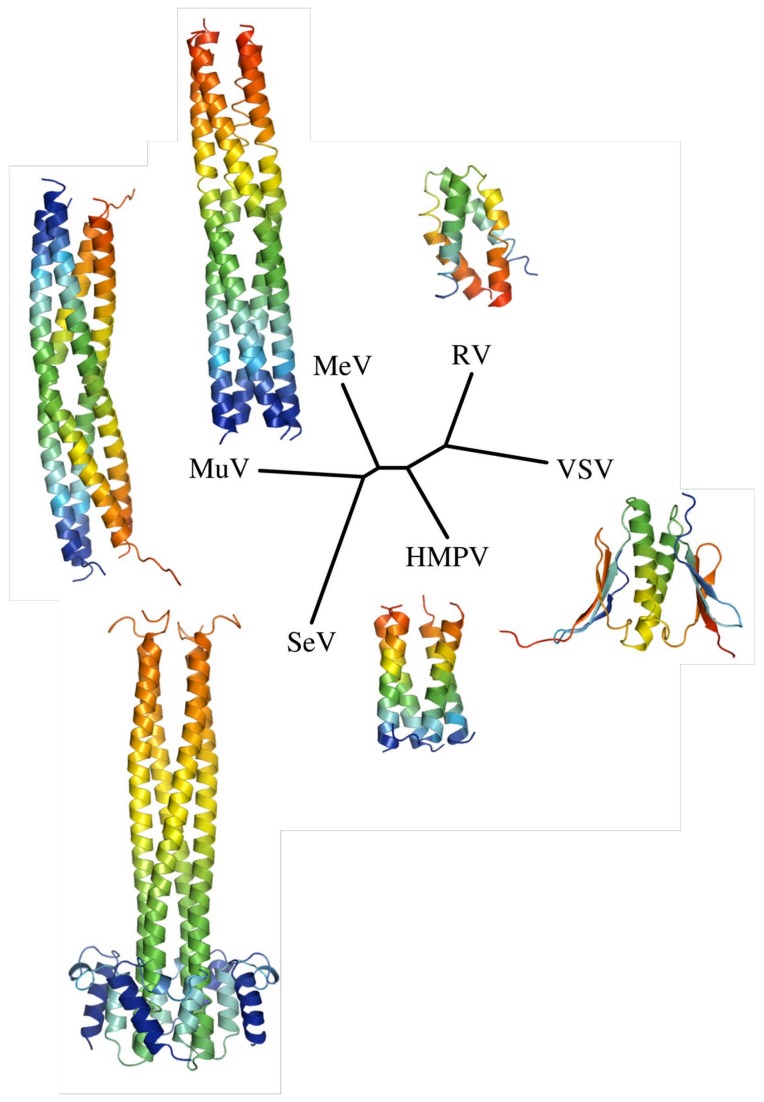
Structure-based phylogenetic tree of *Mononegavirales*P proteins. The tree was built by aligning the experimentally determined structures of Sendai virus (SeV) (PDB ID: 1EZJ), Measles virus (MeV) (PDB ID: 3ZDO), Mumps virus (MuV) (PDB ID: 4EIJ), Rabies Virus (RV) (PDB ID: 3L32), Vesicular stomatitis virus (VSV) (PDB ID: 2FQM) and HMPV P oligomerization domains using SHP [43]. The obtained evolutionary distances were used to draw a tree in PHYLIP [77].

P proteins from *Mononegavirales* are large modular proteins that are characterized by extensive IDRs of variable lengths containing multiple MoRE, a central oligomerization domain and, in some but not all viruses, a stable C-terminal domain [13,38–42]. Because P protein sequences vary greatly in length (from 241 residues in RSV P to 709 residues in Nipah virus P), and have diverged beyond remote homology detection, it is difficult to compare P proteins from different families, or sometimes even different genera. However, the available structures of P oligomerization domains allow us to determine phylogenetic relationships from structural alignments. Figure 9 shows a phylogenetic tree of the crystal structures of tetrameric *Paramyxoviridae* P and dimeric *Rhabdoviridae* P oligomerization domains, built using the structure homology program SHP [43]. Interestingly, the tree obtained from P_ced_ structures is similar to trees built based on large numbers of sequences from more conserved proteins such as N, M, F and L [44]. The tree highlights the structural divergence of P protein oligomerization domains across evolution, with more than three-fold variation in domain length across the *Paramyxoviridae*. The short tetrameric coiled coil from HMPV P clusters in a separate branch from SeV/MuV/MeV and RV/VSV, emphasizing the pertinence of its classification as a separate subfamily, *Pneumovirinae*. 

### The combined use of ab initio modeling with small angle X-ray scattering and molecular dynamics appears to be a promising approach for solving the structure of small homo-oligomericprotein complexes

The work presented in this study combines several computational techniques commonly used in protein structure prediction to yield a correct model for an unknown protein, applying low resolution information about shape and flexibility derived from SAXS as the sole experimental constraint. The advantages of the method can be summarized in four points (1). The recently developed Rosetta fold-and-dock protocol takes advantage of the reduced conformational space available to homo-oligomeric proteins to predict atomistic models [27]. (2) SAXS can be used to successfully filter out a large proportion of incorrect models, as has been shown in*ab initio* protein structure prediction [44–46] and protein-protein docking [47,48]. (3) The usefulness of SAXS data to identify correct models can be increased by extracting information about protein flexibility and disorder through ensemble analysis [28,30], thus tackling the challenges associated with the modeling of partially unstructured proteins. (4) Classical MDS provide an additional mean of selecting and optimizing correct models and detecting flexible regions[49], while its sampling limitations can be overcome by using fast SBM MDS [29,50]. By combining methods (1) to (4), we obtained a detailed, cross-validated picture of HMPV P_ced_ structure and dynamics in solution, showing that it constitutes a promising approach for protein structure determination. The crystal structure of the core region of P_ced_ comes as a validation of the accuracy of the model, and indirectly confirms the flexibility of the degraded flanking regions. The combined use of *ab initio* modeling, MDS and SAXS-based ensemble optimization constitutes a generally applicable method to predict protein structure, both in the presence of stable, potentially homo-oligomeric domains, transiently structured or completely disordered regions, and should become increasingly useful in the future.

## Material & Methods

### Sequence-based analyses

Computational meta-disorder predictions and conservation scores based on sequence alignment of sequences from *Pneumovirinae* P were calculated following procedures described in [41]. Consensus secondary structure prediction was obtained from the Dismeta webserver [51].

### Protein cloning, expression & purification

The region of the HMPV P gene from strain NL1-00 corresponding to residues 158-237 was amplified by PCR and cloned into pOPINF[52] for expression of P with an N-terminal His6 tag followed by a 3C cleavage site, using a proprietary ligation-independent In-Fusion system (Clontech), following standard procedures. The integrity of the cloned construct was checked by nucleotide sequencing.

The His6-3C-P158-237 construct was expressed in Rosetta2™ *E. coli* cells by overnight incubation under shaking at 17°C following 1 mM IPTG induction of 1 l terrific broth in presence of appropriate antibiotics. Cells were harvested by centrifugation (18°C, 20 min, 4000x g). The resulting cell pellets were resuspended in 20 mMTris, pH 7.5, 150 mM NaCl, 8 M urea. Cells were lyzed by sonication, and the lysate was centrifuged for 45 min at 4°C and 50000x g to remove cell debris. The supernatant was filtered (0.45 μm filter) and loaded on a column containing 2 ml of pre-equilibrated Ni-NTA Agarose (QIAGEN). After extensive washes, the protein was eluted in 20 mMTris, pH 7.5, 150 mM NaCl, 400mM imidazole. The protein was then subjected to size exclusion chromatography on a S200 column equilibrated in 20 mM Tris, pH 7.5, 1 M NaCl. The His6 tag was removed by addition of 3C protease at 4°C for 72h. The cleaved product was further purified through reverse Ni-NTA purification to remove His-tagged 3C protease followed by an additional gel filtration step in 20 mM Tris, pH 7.5, 150 mM NaCl. The protein was concentrated using a Millipore concentration unit (c/o 10 kDa).

### Small angle X-ray scattering experiments

Small angle x-ray scattering measurements of P_ced_ (residues 158 to 237) were performed at the BM29 beamline in the European Synchrotron Radiation Facility (ESRF), Grenoble, France. Data was collected at 20°C, a wavelength of 0.0995 nm and a sample-to-detector distance of 1 m. 1D scattering profiles were generated and blank subtraction was performed by the data processing pipeline available at BM29 at the ESRF. 

### Computational modeling of P_ced_

The amino-acid sequences of P residues 155-241 or 156-237were used as input to the Rosetta fold-and-dock protocol with the default recommended parameters [27,53,54]. 2 x 30,000 models were generated and ranked using the Rosetta scoring function. In a second step, models were fitted to experimental SAXS data using CRYSOL[55]to yield the agreement between theoretical and experimental profile χ_exp_. χ_exp_ values were then used to discard incorrect models based on an arbitrary threshold (χ_exp_>1.3). 

### Molecular dynamics simulations and ensemble optimization

All classical MDS were performed using the GROMACS 4 software package [56] and the AMBER99SB-ILDN* force field [57,58]. At the beginning of each simulation, the protein was immersed in a box of SPC/E water. A minimum distance of 1.0 nm was applied between any protein atom and the edges of the box. Sodium ions were added to reach neutrality. Long range electrostatics were treated with the particle-mesh Ewald summation [59]. Bond lengths were constrained using the P-LINCS algorithm [60]. Hydrogens were treated as virtual sites [61], enabling an integration time step of 5fs. The v-rescale thermostat [62]and the Parrinello–Rahman barostat[63] were used to maintain a temperature of 300 K and a pressure of 1 atm. Each system was energy minimized using 1,000 steps of steepest descent and equilibrated for 200 ps with restrained protein heavy atoms before the beginning of the production simulation. For each system, two independent production simulations were obtained by using different initial velocities. The aggregated simulation time was ~4.15 µs (Table 2). Calculation of root mean square deviations (RMSD) and root mean square fluctuations (RMSF) were carried out using GROMACS routines. Snapshots were extracted every 500 ps, resulting in a pool of ~8,300 models. 

In order to obtain a more complete sampling of the IDRs, in particular those located between residues 195 and 237, model 1 was simulated using an atomistic coarse-grained structure-based model (SBM) [29,50]. Model 1 was selected because of the higher stability of the α-helical fold adopted by residues 195 to 220, as shown by its low RMSF (Figure 4), and also its higher frequency of selection in optimized ensembles (not shown). Two additional systems were simulated with either residues 220 to 237, or residues 195 to 237 in extended starting conformations, allowing fast sampling of the IDRs motions. 2000 snapshots were extracted from each simulation, yielding 4,000 additional models to the pool. 

For each model from the pool ensemble (~12,300 models), theoretical SAXS patterns were calculated with the program CRYSOL [55] and ensemble fitting was performed with GAJOE [28]. The number of models in the selected ensemble was varied from 1 to 20 in order to determine the size of the minimal ensemble required to describe the data.

### Crystallization and data collection

Crystallization was carried out via the vapor diffusion method using a Cartesian Technologies pipetting system [64]. The P158-237 construct crystallized after ~142 days in 25 % PEG 3350, 100 mM HEPES pH 7.5 at 20°C. Crystals were frozen in liquid nitrogen after being soaked in a mother liquor solution supplemented with 25% glycerol. Diffraction data was recorded on the I04 beamline at Diamond Light Source, Didcot, UK.

### Structure determination and refinement

Anisotropic diffraction data to a resolution of 3.1Å were indexed and integrated using XDS [65] and scaled with SCALA [66] as implemented in the program xia2 [67]. The structure was determined by molecular replacement using P_core_ residues from model 1 as a search model in PHASER [68]. The solution was subjected to repetitive rounds of restrained refinement in PHENIX [69] and Autobuster [70] and manual building in COOT [71]. Eight-fold non crystallographic local structure similarity restraints [72] were used throughout refinement and TLS parameters were included in the final round of refinement. The CCP4 program suite [73] was used for coordinate manipulations. The structures were validated with Molprobity [74]. Refinement statistics are given in Table 3, and final refined coordinates and structure factors have been deposited in the PDB with accession code 4BXT.

### Structure analysis

All the structure-related figures were prepared with the PyMOL Molecular Graphics System (DeLano Scientific LLC). Protein interfaces were analyzed with the PISA webserver [75]. Structural alignments were calculated using PyMOL and SHP [43].
